# Comprehensive Assessment of Left Atrial Function: The Emerging Role of Cardiac Magnetic Resonance Feature Tracking

**DOI:** 10.3390/jcdd12090337

**Published:** 2025-09-02

**Authors:** Javier Solsona-Caravaca, Alessandro Giustiniani, Eduard Ródenas-Alesina, Laura Galian-Gay, Ruperto Oliveró, Filipa Valente, Guillem Casas, Gisela Teixidó-Turà, Nuria Vallejo, Rubén Fernández-Galera, Víctor González-Fernández, Pablo Escribano-Escribano, Axel Hernández-Pineda, Ignacio Ferreira-González, José Fernando Rodríguez-Palomares

**Affiliations:** 1 Cardiology Department, Vall d’Hebron Hospital Universitari, Vall d’Hebron Barcelona Hospital Campus, Passeig Vall d’Hebron 119-129, 08035 Barcelona, Spain; javier.solsona@vallhebron.cat (J.S.-C.); laura.galian@vallhebron.cat (L.G.-G.); rupertocarlos.olivero@vallhebron.cat (R.O.); pablo.escribano@vallhebron.cat (P.E.-E.);; 2 Centro de Investigación Biomédica en Red Enfermedades Cardiovasculares (CIBERCV), Instituto de Salud Carlos III, 28029 Madrid, Spain; 3 Cardiovascular Diseases, Vall d’Hebron Institut de Recerca (VHIR), Vall d’Hebron Hospital Universitari, Vall d’Hebron Barcelona Hospital Campus, Passeig Vall d’Hebron 119-129, 08035 Barcelona, Spain; 4 Department of Medicine, Universitat Autònoma de Barcelona, 08193 Bellaterra, Spain; 5 Centro de Investigación Biomédica en Red de Epidemiología y Salud Pública (CIBERESP), Instituto de Salud Carlos III, 28029 Madrid, Spain

**Keywords:** left atrial strain, cardiovascular magnetic resonance, feature tracking, risk stratification, valvular heart disease, cardiomyopathy

## Abstract

Traditional volumetric parameters fall short of capturing the complex, phasic nature of atrial function. In contrast, atrial strain has become recognized as a sensitive, non-invasive imaging marker that enables earlier detection of myocardial dysfunction, refined risk stratification, and individualized therapeutic decision-making across a wide range of cardiovascular diseases. Cardiovascular magnetic resonance feature tracking (CMR-FT) has emerged as a robust imaging technique for evaluating atrial strain, offering high spatial resolution, high reproducibility, and independence from acoustic window limitations. Despite its promise, the routine clinical adoption of CMR-FT atrial strain remains limited. Key barriers include intervendor variability in strain values, the absence of standardized post-processing protocols, the lengthy acquisition times inherent to CMR studies, and the time required for post-processing atrial strain analysis. Overcoming these barriers is crucial to facilitate the integration of atrial strain assessment into routine clinical CMR protocols, particularly in patients with heart failure, valvular disease, or cardiomyopathy who undergo imaging for diagnostic or prognostic evaluation.

## 1. Introduction

In recent years, evaluating the performance of the left atrium (LA) has become increasingly important for diagnosing and prognosing various diseases, as it is recognized as a key marker of adverse cardiovascular events [1,2]. Traditionally, assessments were limited to morphological analyses based on diameters and volumes [2]. However, LA enlargement alone does not fully capture the dynamic changes occurring throughout the cardiac cycle [3].

Global longitudinal left atrial strain (LAS) enables the detection of LA functional impairment before anatomical remodeling occurs. Furthermore, LAS assessment may allow the detection of several cardiovascular diseases at an earlier stage, thereby aiding clinicians in their decision-making and improving patient outcomes [3].

While transthoracic echocardiography (TTE) with speckle tracking has been widely used for quantifying LAS, there is a growing emphasis on analyzing LA deformation using cardiac magnetic resonance (CMR) [2].

This review aims to summarize the current understanding of LAS evaluation with CMR feature tracking (CMR-FT) and explore its clinical applications, including its prognostic role.

## 2. Left Atrial Dynamics in the Cardiac Cycle: Physiology and Imaging Assessment

### 2.1. Understanding the Physiological Role of Left Atrial Function

The LA, due to its complex anatomical and physiological connections, fulfils various electrical and hemodynamic roles during the cardiac cycle. Particularly, the rheological interplay between the LA and left ventricle (LV) is pivotal for a physiological and efficient LV filling [4], with the two chambers linked together as a functional unit.

**Figure 1:**
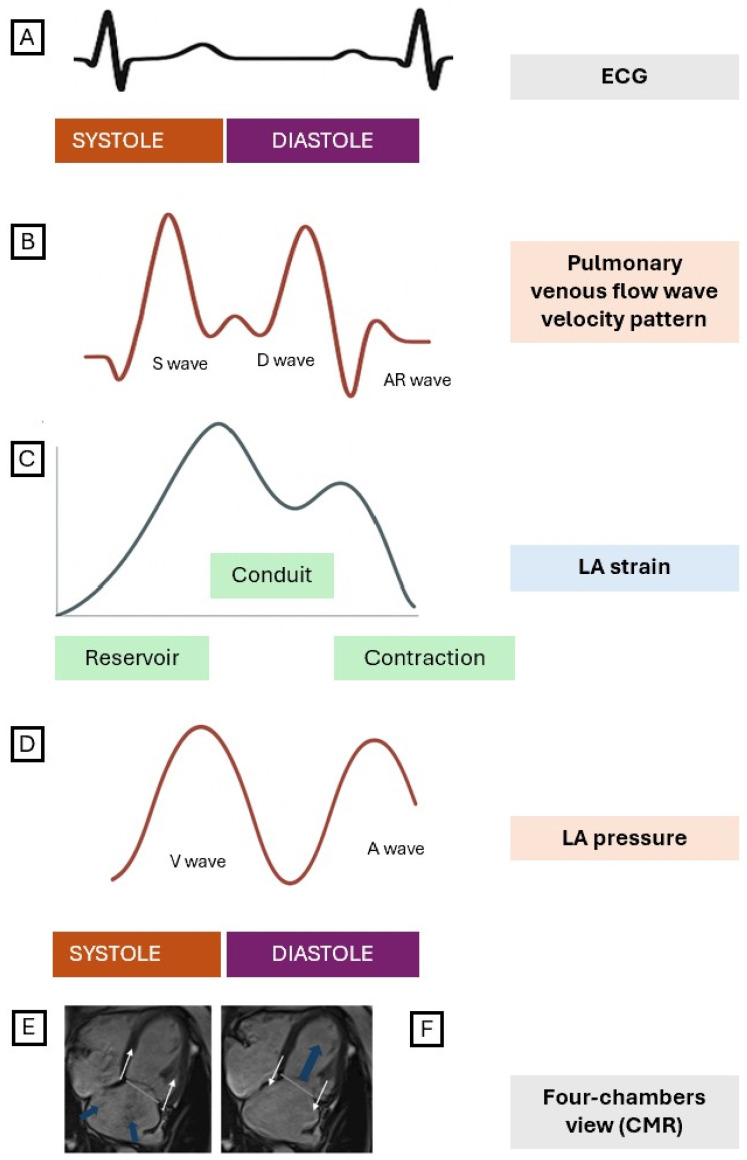
Interaction between mitral annular motion, pulmonary venous flow, LA, and LV function. (**A**) shows a standard ECG tracing used as a temporal reference for the cardiac cycle. (**B**) illustrates the Doppler flow velocity pattern of pulmonary venous return into the LA, which includes three characteristic waves: the S wave, reflecting pulmonary venous flow into the LA during LV systole; the D wave, indicating flow during early LV diastole; and the AR wave, representing retrograde flow into the pulmonary veins during LA contraction. (**C**) shows the LA strain curve, which comprises three functional phases: the reservoir phase, the conduit phase, and the contraction phase. (**D**) displays the LA pressure waveform throughout the cardiac cycle. The V wave reflects LA filling during ventricular systole, while the A wave corresponds to the pressure increase during LA contraction. (**E**) During the reservoir phase, the descent of the mitral annulus (white arrows) facilitates pulmonary venous inflow into the LA (blue arrows). (**F**) The conduit phase is promoted by the mitral annulus moving away from the apex (white arrows), which facilitates LV filling (blue arrow). LA, left atrium; LV, left ventricle; ECG, electrocardiogram; S, systolic; D, diastolic; AR, atrial reversal; CMR, cardiac magnetic resonance.

Blood flow within and along the LA can be roughly summarized into three main phases (Figure 1):

- Reservoir phase.

During the LV systole, the mitral plane is pulled toward the cardiac apex, stretching the LA, which is tightly anchored posteriorly by the pulmonary veins [5]. Thus, it favors atrial filling due to the pressure drop within the chamber. Pulmonary flow progressively slows down as LA pressure rises alongside atrial volume, which peaks at the start of LV diastole during isovolumic relaxation, just before the mitral valve opening [3].

- Conduit phase.

As the mitral valve opens, the blood flows toward the LV cavity driven by the atrioventricular (AV) pressure gradient, which results from the increased LA pressure and the simultaneous decline in LV pressure, conditioned by myocardial lusitropy and ventricular wall compliance [3]. Concomitantly, the mitral plane rises back up toward the LA posterior wall, with atrial recoil and a decrease in its tension after stretching [4]. A physiological vortex within the LA facilitates the efficient transfer of blood from the pulmonary veins to the atrioventricular inflow, which is oriented nearly perpendicularly to them [6,7].

- Active contraction phase.

In late diastole, atrial contraction leads to a final surge in LA pressure, pumping part of the remaining atrial blood volume toward the LV and pulling back the mitral annulus. The degree of atrial contraction to overall ventricular filling depends on numerous factors such as heart rate, heart rhythm, atrial electromechanical coupling, atrial volume, and ventricular stiffness [8].

### 2.2. From Physiology to Imaging: Assessment of LA Function

By employing various tracking techniques throughout the cardiac cycle, the deformation of the endocardial wall can be quantified using Lagrangian strain, which represents the percentage change in length relative to a reference atrial contour. The strain value is either positive or negative, depending on whether the atrial wall is stretching or contracting, respectively [9].

The most widely adopted parameter is the global longitudinal strain, which quantifies the relative length change of the endocardial border along its tangential axis in long-axis views [9]. Due to technical limitations and the lack of robust data, segmental strain and wall deformation in other directions (i.e., circumferential and radial strain) are not currently endorsed by consensus panels for clinical reporting [4,9].

To calculate LAS, a reference setpoint of atrial dimension is needed. The Task Force recommends that the default baseline reference for LA strain curves should be end-diastole (ED). The LAS curve characteristically exhibits a first peak (i.e., LAS reservoir), followed by a variable decline to a plateau in diastasis (i.e., LAS conduit) and a second drop during the atrial contraction (i.e., LAS contraction or booster) [9].

The three main reported parameters of the LAS curve, which reflect the distinct phases of atrial physiology, are defined by the difference between two specific points on the LAS curve (Figure 1) [9]:

- Reservoir phase: Difference between LAS peak at MVO and ED frame (positive value).
- Conduit phase: Difference between LAS value at the onset of atrial contraction and MVO (negative value).
- Contraction phase: Difference between LAS value at ED frame and at the onset of atrial contraction (negative value).

LAS is customarily calculated on a non-foreshortened standard LV long-axis view, defined as a plane traversing both the LV apex and the mitral valve orifice, most commonly the four-chamber view or two-chamber view. However, this methodology may result in artificial foreshortening of the LA, potentially overestimating LAS [10].

Some studies have further expanded on better modeling of the LA chamber, either by incorporating the three-chamber view into a triplane model [11] or by using a fully three-dimensional (3D) imaging technique [10]. Nevertheless, until more robust evidence supports their widespread clinical use, the currently recommended biplane approach—using two- and four-chamber views—remains the standard in clinical practice due to its feasibility and proven prognostic value [4,9].

### 2.3. Clinical and Hemodynamic Determinants of LAS

LAS is determined primarily by two main factors: the absolute longitudinal atrial lengthening and the ED atrial dimension. ED atrial size serves as the baseline scale and inevitably heavily affects all derived LAS values [12]. Therefore, a larger ED dimension leads to lower LAS measurement despite similar longitudinal mitral valve excursion.

The primary contributor to LAS is the AV plane displacement, as the pulmonary veins anchor the posterior LA wall, and it remains relatively fixed throughout the cardiac cycle [5]. LAS is, therefore, sensitive to parameters influencing the AV plane mobility, including LV contraction [13], LA parameters of stiffness and pressure [14], and the ratio between LA to LV volumes, which modulates the association between LAS and LV global longitudinal strain (LV GLS) [13].

LA contraction influences LAS in two key aspects: by directly contributing to annular motion and by reducing the overall atrial ED dimension. Therefore, in conditions with impaired atrial contractility, such as atrial fibrillation (AF), both the conduit and contraction phases are diminished or abolished. This results in a higher ED atrial dimension (due to incomplete emptying) and a lower overall LAS peak [15].

Advancing age has been correlated with a decline in the LAS reservoir and conduit [16]. However, a relative increase in LA contraction strain has also been observed in older individuals, reflecting the lower contribution of passive ventricular filling in a stiffer LV [17].

## 3. Technological Innovations in LA Strain Assessment: CMR-FT as a Novel Tool

### 3.1. Comparative Evaluation of LA Function: TTE Versus CMR—Strengths and Limitations of CMR

Due to its widespread availability, LA phasic volumes and strain measured by TTE are the primary methods for assessing LA function [4]. However, TTE has several limitations (Table 1), including a suboptimal field of view in patients with poor acoustic windows and high interobserver variability [3].

On the other hand, CMR is considered the gold standard for volumetric and functional ventricular assessment and provides an effective alternative for LA evaluation. Not only is it not influenced by the patient’s habitus, but it also offers excellent spatial resolution and an adjustable field of view [3]. Furthermore, CMR-FT-based LAS analysis offers greater reproducibility and lower intra- and inter-observer variability compared to TTE [3,18].

Additionally, CMR has a flexible approach tailored to the patient, with the possibility of expanding atrial assessment beyond morpho-volumetric and deformation measures. Using specific CMR sequences, data on pulmonary vein patterns, atrial fibrosis [4], peri-atrial fat [19], and even intra-atrial overall flow [7] can also be provided.

Despite its apparent benefits, several limitations of CMR in assessing LA function must be recognized (Table 1). First, CMR-FT is inherently affected by factors that reduce the wall-to-blood intensity gradient, such as pulmonary vein flow artifacts, the presence of interatrial or mitral valve devices, severe calcifications, or the use of cine sequences with lower wall-to-blood contrast [20].

Second, the conventional multi-beat acquisition for standard cine imaging is susceptible to beat-to-beat variation in heart motion, particularly in patients with arrhythmias. However, recent advances in scanner technology have enabled specific single-at-scan acquisitions, allowing for a more reliable LAS quantitative assessment in these challenging cases [21,22].

Third, a routine CMR acquisition offers a much lower temporal resolution than TTE. This may impact the ability to assess subtle nuances of atrial mechanics, as a higher frame rate is associated with higher LAS values [23]. However, the impact on LAS measurements in the standard protocol, typically with approximately 30 frames per beat, appears to be relatively modest, whereas it might be more pronounced for LA strain rates [24].

Finally, the major drawback is the limited availability and higher cost of a CMR scan compared to TTE [3]. Thus, while strain assessment could become part of a routine CMR exam, its applicability to the broader public will remain more limited than that of a TTE.

**Table 1:** Comparative evaluation of LA function: TTE versus CMR.

	TTE	CMR
Availability	Widely available; routine clinical use [4].	Limited availability, higher cost, and less widespread use [3].
Time for acquisition/analysis	Short.	Longer.
Image quality	Limited by the acoustic window and patient habitus.	Not affected by body habitus; excellent image quality [3].
Spatial resolution	Lower spatial resolution compared to CMR.	Excellent spatial resolutions and an adjustable field of view [3].
Temporal resolution	Superior frame rate; associated with higher LAS values [23].	Lower.
Susceptibility to Artifacts	Affected by the acoustic window.	Affected by valve or interatrial devices, severe calcifications, and pulmonary vein flow artifacts [20].
Susceptibility to arrhythmias	Lower.	Higher, mitigated by advanced single-scan acquisitions [21,22].
Reproducibility of LAS assessment	Lower reproducibility; operator dependent.	Superior reproducibility and lower interobserver variability [3,18].
Atrial tissue characterization	Not available.	Possible assessment of LA fibrosis (4) and peri-atrial fat [19].
Clinical role	Primary modality for routine LA functional assessment.	A complementary tool with an expanding role in advanced evaluation.

LA, left atrial; TTE, transthoracic echocardiography; CMR, cardiac magnetic resonance; LAS, longitudinal left atrial strain.

### 3.2. LAS Analysis Using CMR-FT

The use of standard LV-centered views for LAS assessment is advantageous because these views are already acquired as part of routine cardiac imaging protocols for LV function [25], so no additional image acquisition is required.

LAS assessment in routine CMR is primarily based on FT algorithms applied to routinely acquired cine images in the long-axis view. FT relies on intrinsic image features, such as voxel texture intensity variation and sharp intensity transitions between tissue interfaces (e.g., between the myocardium and the blood pool) [26].

The subsequent motion of these features is estimated across the cardiac cycle, usually using optical flow models, which track their displacement frame by frame [20].

Many vendors offer their commercially available FT-analysis software, which includes their specific proprietary algorithm. Most of them can automatically or semi-automatically determine the blood–wall interface and the reference frames for end-diastole and end-systole. Some vendors offer specific interfaces for LA strain assessment, while others utilize their established FT model for LV strain, adapting it to atrial morphology, using endocardial or even epicardial tracking.

The user will generally need to:

- Select the image or validate the software image selection for the LAS measurement.
- Choose a reference image frame (usually end-systole or end-diastole).
- Manually draw or check the automatic endocardial border (or also epicardial border if required) in the reference frames.
- Activate the FT algorithm and assess wall tracking quality along the cine sequence.

### 3.3. Reference Values for LAS Using CMR-FT

Magnetic field strength [27] and the MR scanner vendor [28] appear to have little or no influence on determining FT strain values. However, the proprietary FT-tracking algorithm is a recognized source of heterogeneity and systematic variation in measurements [28,29]. Consequently, LAS measurements may be affected by changes in post-processing software vendors or even by the same vendor’s software version update if an explicit change is introduced in the FT underlying algorithm.

A recent meta-analysis, pooling FT-based LAS values from 747 normal subjects across ten different studies with varying post-processing software vendors, reported the distribution of normal values for all three LAS values [28]. These data were adopted by the latest consensus statement on multimodality imaging of the LA [4]. The reference ranges for LA strain values, as assessed by CMR, are presented in Table 2.

Due to the differences between CMR and TTE in LA strain assessment, as well as the variability among different CMR software platforms, sequential evaluations in the same patient should be performed using the same imaging modality and software to ensure consistency and reliability [2].

## 4. LA Strain Assessment by CMR-FT: Diagnostic Value and Prognostic Implications

Recent studies have shown that evaluating atrial function through CMR-FT can detect early LA dysfunction in patients with cardiovascular risk factors or established heart disease. This approach provides incremental diagnostic value by enhancing risk stratification and offering predictive insights into the occurrence of major adverse cardiovascular events (MACE) and long-term prognosis [18].

### 4.1. Identifying Patients at Risk of Cardiovascular Disease

Lower LAS assessed by CMR-FT independently predicts the onset of heart failure (HF) in multiethnic, asymptomatic individuals [30].

In patients with hypertension, impaired LAS reservoir and conduit are frequently observed, even without LA dilatation or LV hypertrophy, suggesting that functional abnormalities precede structural remodeling [31]. Reduced LAS may serve as an early marker for initiating antihypertensive treatment and enhancing cardiovascular risk stratification.

### 4.2. Atrial Fibrillation

LAS components can predict the onset of AF in elderly patients with stroke risk factors but no prior history of AF [32,33]. Notably, patients with low LAS reservoir (<33%) had a significantly higher risk of AF, with an incidence rate of 14.5 compared to 9.8 events per 100 person-years in those with higher LAS [33]. Consequently, LAS assessment may aid in guiding early anticoagulation decisions in patients at risk without documented AF.

In patients with established AF, those with persistent AF exhibit a larger maximum end-systolic LA volume index (LAVI), a greater extent of LA late gadolinium enhancement (LGE), and significantly reduced LAS compared to those with paroxysmal AF [34]. Among patients with paroxysmal AF who are in sinus rhythm, LAS is reduced compared to healthy controls [35].

### 4.3. Heart Failure with Preserved Ejection Fraction

Right heart catheterization with exercise stress remains the reference standard for diagnosing HF with preserved ejection fraction (HFpEF). However, due to the invasive nature of the test, CMR exercise imaging has emerged as a promising non-invasive diagnostic alternative [36].

The impairment of the LAS conduit observed in patients with HFpEF may reflect a compromised early coupling of ventricular filling. Notably, a strong correlation has been demonstrated between the LAS conduit and peak oxygen uptake during cardiopulmonary exercise testing [37].

Compared to pre-capillary pulmonary hypertension (PH), patients with HFpEF and post-capillary PH exhibit lower LAS. Atrial assessment using CMR-FT can accurately distinguish between post- and pre-capillary PH [38].

### 4.4. Heart Failure with Reduced Ejection Fraction

#### 4.4.1. Dilated Cardiomyopathy

**Figure 2:**
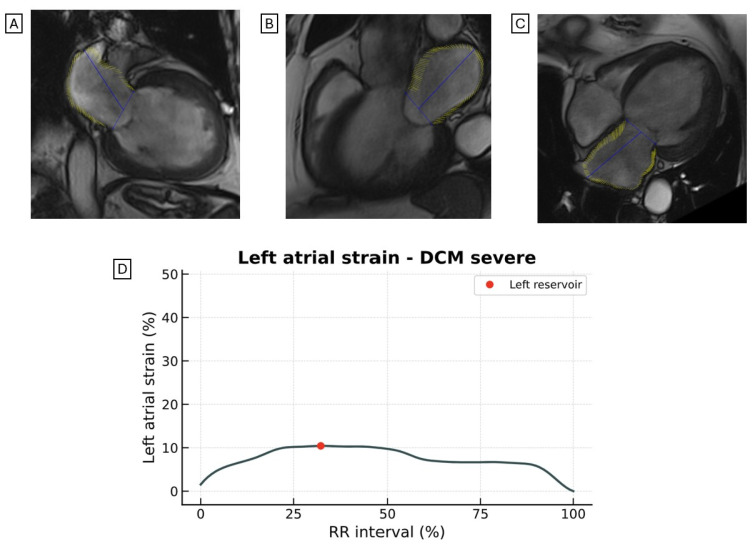
Representative images of LAS assessed by CMR-FT from the (**A**) two-chamber, (**B**) three-chamber, and (**C**) four-chamber views in a patient with DCM. The LV exhibits a spherical morphology, marked dilation, and severely reduced LVEF. (**D**) Quantitative analysis reveals a markedly reduced LAS. LAS, longitudinal left atrial strain; CRM-FT, cardiac magnetic resonance feature tracking; DCM, dilated cardiomyopathy; LV, left ventricle; LVEF, left ventricular ejection fraction.

**Figure 3:**
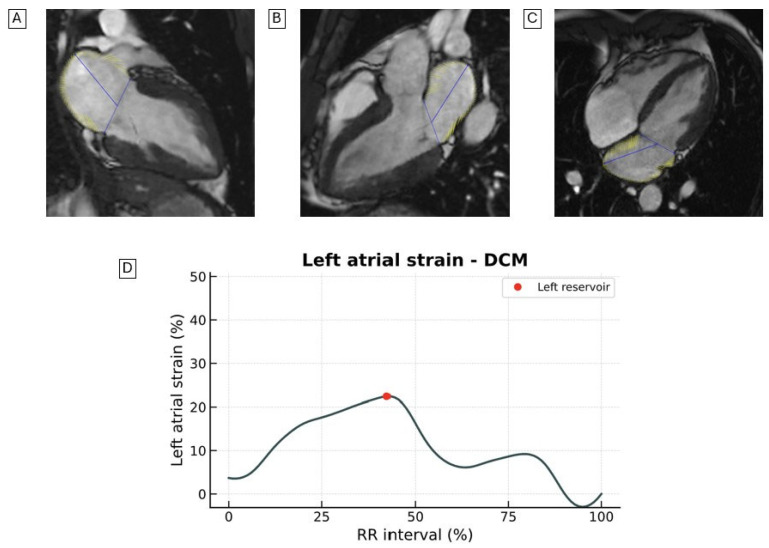
Representative images of LAS assessed by CMR-FT from the (**A**) two-chamber, (**B**) three-chamber, and (**C**) four-chamber views in a patient with DCM. The LV demonstrates mild dilation and mildly reduced LVEF. (**D**) Quantitative LAS curves are shown. LAS, longitudinal left atrial strain; CRM-FT, cardiac magnetic resonance feature tracking; DCM, dilated cardiomyopathy; LV, left ventricle; LVEF, left ventricular ejection fraction.

LAS is reduced in patients with HF (Figure 2 and Figure 3) and is more impaired in HFrEF patients than in those with HFpEF [39].

Among patients with DCM, Chirinos et al. demonstrated that the LAS conduit (hazard ratio, 0.66; *p* = 0.004) and LAS reservoir (hazard ratio, 0.68; *p* = 0.005) independently predict the risk of HF-related admissions or death [39].

Other studies have confirmed that LAS measured by CMR-FT is a strong prognostic marker [40,41], superior to classic imaging parameters such as LV GLS, LV ejection fraction (LVEF), LAVI, and LV LGE [42,43]. An LAS conduit value < 12% identifies patients at higher risk of mortality and HF hospitalization [42].

Recently, there has been growing interest in characterizing LV and LA remodeling in patients with HF undergoing novel pharmacological therapies [44,45,46]. In this context, LAS assessment using CMR provides a unique, non-invasive tool for evaluating these structural and functional changes, which may serve as an early marker of treatment response.

#### 4.4.2. Takotsubo Syndrome

The pathophysiology of Takotsubo syndrome involves transient impairments in the LAS reservoir and LAS conduit, alongside enhanced LAS contraction [47].

LAS assessed by CMR-FT is a superior predictor of mortality after Takotsubo syndrome compared to LVEF and LAVI [47]. In a multicenter study [47], the area under the curve (AUC) for LAS predicting mortality was 0.71, outperforming LVEF (AUC 0.59) and LAVI (AUC 0.62).

### 4.5. Cardiomyopathies

#### 4.5.1. Excessive Trabeculation of the Left Ventricle

**Figure 4:**
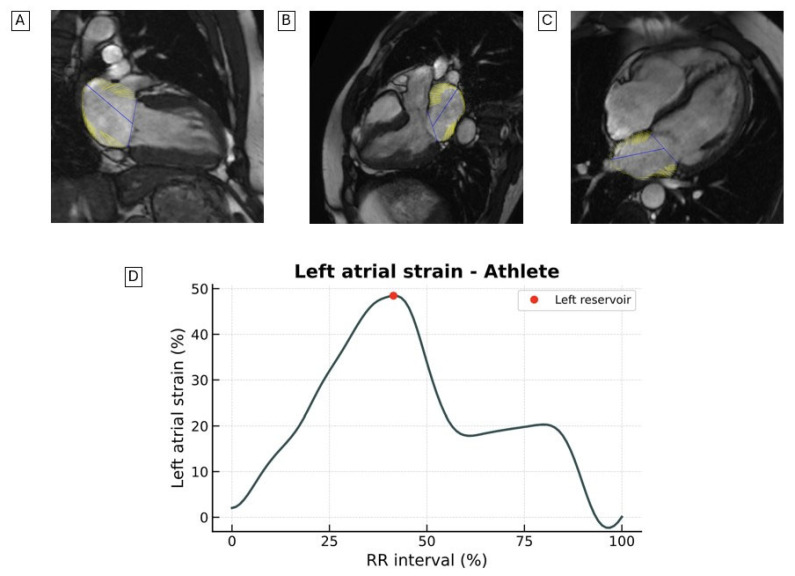
Representative images of LAS assessed by CMR-FT from the (**A**) two-chamber, (**B**) three-chamber, and (**C**) four-chamber views in a young athletic patient with ETLV. (**D**) Quantitative LAS strain curves are shown. The patient exhibits no evidence of hypertrophy, normal cardiac chamber volumes, a preserved LVEF, absence of LGE, and maintained LAS—findings consistent with physiological adaptations seen in the athlete’s heart. LAS, longitudinal left atrial strain; CRM-FT, cardiac magnetic resonance feature tracking; ETLV, excessive left ventricular trabeculation; LVEF, left ventricular ejection fraction; LGE, late gadolinium enhancement.

LAS may help differentiate physiological hypertrabeculation from subclinical cardiomyopathy in patients with excessive left ventricular trabeculation (ETLV) (Figure 4).

Moreover, LAS may serve as a valuable tool for improving risk stratification in patients with ETLV [48,49]. In this context, Casas et al. showed that among ETLV patients with preserved LVEF (≥50%) and no LGE, those with an LA reservoir strain <22.6% had a MACE incidence >20%, compared to <1% in those with LAS values above this threshold [48].

#### 4.5.2. Hypertrophic Cardiomyopathy

**Figure 5:**
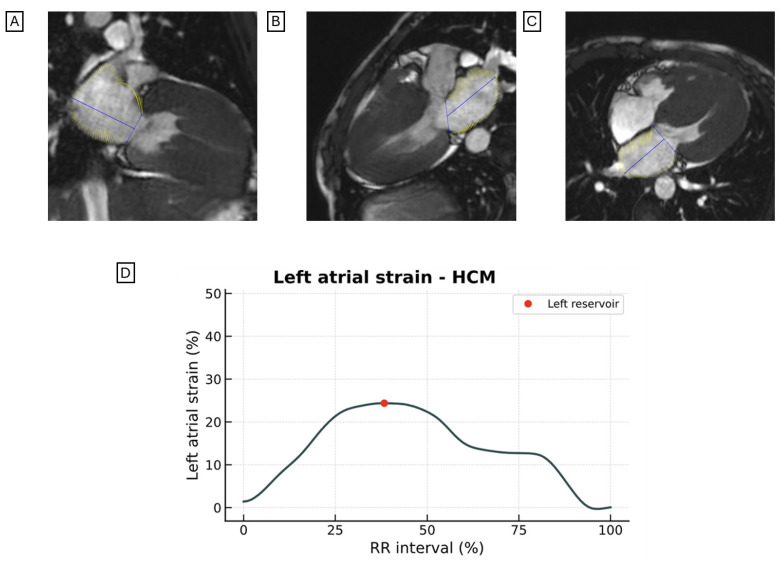
Representative images of LAS assessed by CMR-FT from the (**A**) two-chamber, (**B**) three-chamber, and (**C**) four-chamber views in a patient with massive HCM. (**D**) Quantitative LAS curves are presented. LAS, longitudinal left atrial strain; CRM-FT, cardiac magnetic resonance feature tracking; HCM, hypertrophic cardiomyopathy.

The LAS reservoir is significantly impaired in HCM patients (Figure 5) even in those with normal LAVI and normal LV filling pressures (16.7 ± 7.1% in HCM vs. 27.6 ± 8.3% in healthy controls, *p* < 0.001) [50]. Multiple studies have consistently demonstrated that both the LAS reservoir and conduit are impaired in HCM, while the LAS contraction is relatively preserved [51]. The LAS reservoir and conduit predict MACE in patients with HCM [50,52,53], outperforming conventional markers such as LA size and the presence of LV LGE [53]. An LAS contraction ≤ 8.9% is an independent predictor of a composite endpoint that includes HF, cardiovascular death, and new-onset AF [54]. Also, an LAS contraction ≤ 8% and an LAS reservoir ≤ 18% have been identified as independent predictors of new-onset AF in patients with HCM, highlighting the potential utility of LAS in guiding prophylactic anticoagulation strategies in this population [55].

#### 4.5.3. Cardiac Amyloidosis

LAS parameters derived from CMR-FT have been investigated as potential diagnostic tools for cardiac amyloidosis (CA) to distinguish its clinical subtypes and differentiate it from other hypertrophic phenotypes [56].

**Figure 6:**
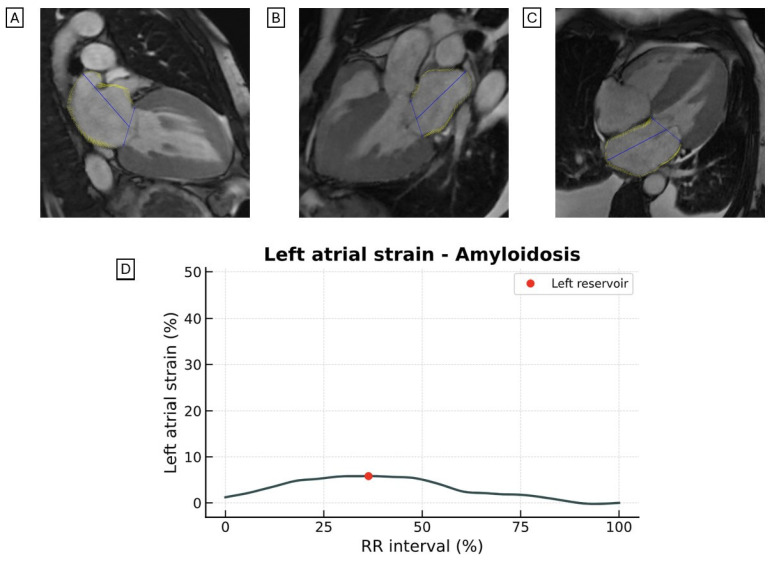
Representative images of LAS assessed by CMR-FT from the (**A**) two-chamber, (**B**) three-chamber, and (**C**) four-chamber views in a patient with CA. (**D**) LAS curves demonstrate markedly reduced strain values in CA compared to those observed in patients with HCM. LAS, longitudinal left atrial strain; CRM-FT, cardiac magnetic resonance feature tracking; CA, cardiac amyloidosis; HCM, hypertrophic cardiomyopathy.

No significant differences were observed in LAS between patients with amyloid light-chain (AL) and transthyretin (ATTR) amyloidosis. However, LAS was significantly lower in CA (Figure 6) than in HCM (LAS reservoir 8.3 ± 5.6% in CA vs. 16.8 ± 8.3% in HCM, *p* < 0.001; LAS conduit 5.5 ± 4.0% in CA vs. 7.9 ± 4.0% in HCM, *p* = 0.004) [56].

In a cohort of patients with histologically confirmed AL amyloidosis, increasing amyloid burden—quantified by LV LGE and extracellular volume (ECV)—was associated with progressive impairment in all components of LAS [57]. In multivariate analyses, the LAS reservoir, New York Heart Association (NYHA) functional class, and ECV were each independent predictors of survival. Notably, an LAS reservoir <8.6% was associated with an increased risk of mortality [57].

#### 4.5.4. Anderson–Fabry Disease

LA function assessed by CMR-FT reveals early atrial impairment in Anderson–Fabry disease (AFD), even before the development of LV hypertrophy or diastolic dysfunction. Stratification by native T1 mapping shows that patients with reduced T1 values exhibit significantly decreased LAS conduit compared to healthy controls. These findings suggest that LAS may serve as a sensitive marker for early cardiac involvement in AFD, with potential implications for personalized patient management [58].

#### 4.5.5. Systemic Sclerosis

CMR is currently recommended in patients with Systemic sclerosis (SSc) to assess for myocardial inflammation and fibrosis, which may suggest primary cardiac involvement. In patients with SSc, LAS is independently associated with the presence of NYHA class II-IV. While both LAS and LV GLS are linked to all-cause mortality, only LAS offers incremental prognostic value beyond all other established risk factors, including LV LGE [59].

#### 4.5.6. Myocarditis

Myocarditis patients exhibit an impaired LAS reservoir and LAS conduit, which may reflect underlying ventricular diastolic dysfunction [60,61]. In a study including 86 patients, the combination of quantitative CMR-derived strain parameters with the established Lake Louise criteria enhanced diagnostic performance in suspected myocarditis cases, increasing the AUC from 0.78 for the Lake Louise criteria alone to 0.86 when combined with strain analysis [60].

### 4.6. Ischemic Heart Disease and Myocardial Infarction

LAS assessed by TTE or CMR provides incremental diagnostic value over LA volumetric measurements for identifying the presence and severity of diastolic dysfunction in post-myocardial infarction patients. Impaired LAS correlates independently with infarct size and improves the prediction of new-onset AF and congestive HF [62].

In patients with ST-segment elevation myocardial infarction (STEMI), LAS reservoir strain (≤22%) and LAS conduit (≤10%) are independently associated with an increased incidence of MACE. LAS provides additional prognostic value beyond traditional outcome predictors, such as LVEF, microvascular obstruction, and infarct size assessed by LGE [63].

### 4.7. Valvular Heart Disease: Aortic Stenosis

**Figure 7:**
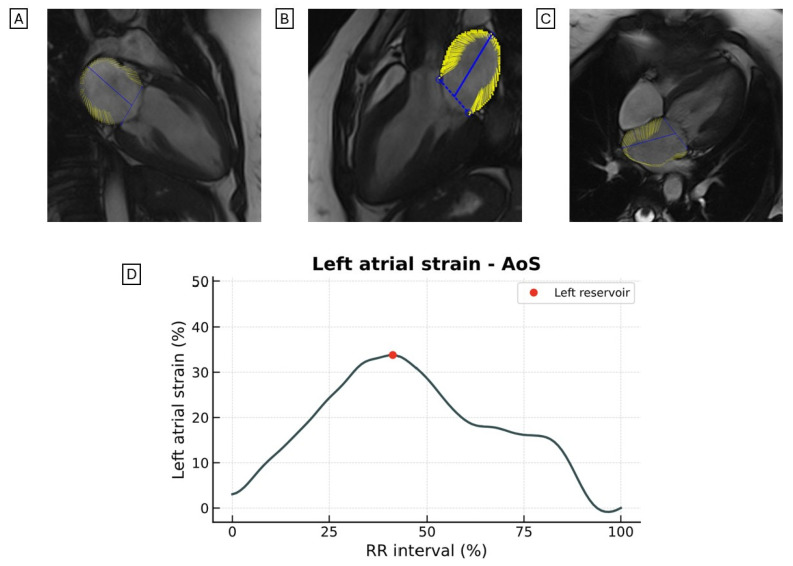
Representative images of LAS assessed by CMR-FT from the (**A**) two-chamber, (**B**) three-chamber, and (**C**) four-chamber views in a patient with AoS. (**D**) Quantitative LAS curves are illustrated. LAS, longitudinal left atrial strain; CRM-FT, cardiac magnetic resonance feature tracking; AoS, aortic stenosis.

Patients with severe aortic stenosis (AoS) often exhibit LA remodeling, which is commonly associated with impaired LAS (Figure 7). An LAS reservoir strain <14.5% has emerged as an independent predictor of mortality and HF in this population [64].

Lange et al. evaluated myocardial remodeling in 40 patients with severe AoS one year after undergoing transcatheter aortic valve replacement (TAVR). Significant improvements were observed in LA functional parameters, which paralleled clinical recovery as evidenced by improvement in NYHA functional class and a reduction in N-terminal pro–B-type Natriuretic Peptide (NT-proBNP) levels. Therefore, LAS analysis using CMR-FT offers valuable insights into the reversal of myocardial remodeling following TAVR in patients with severe AoS [65].

### 4.8. Congenital Diseases

Atrial function is a prognostic indicator in many congenital heart diseases.

In patients with repaired tetralogy of Fallot, LAS is reduced and correlates with the severity of diastolic dysfunction and elevated LV myocardial T1 values [66].

Similarly, in patients with Ebstein anomaly, although LV function is preserved, there is a reduction in LAS, which correlates with the NYHA functional class [67].

In patients with transposition of the great arteries following surgical repair, atrial function is impaired in all cases, regardless of whether they underwent an atrial switch or an arterial switch procedure. Among these, the pulmonary venous atrium, serving as preload for the systemic right ventricle after the atrial switch, is the most significantly altered [68].

Finally, LAS evaluated by CMR-FT is markedly impaired in patients with Fontan physiology. Reduced LAS is associated with lower cardiac index, elevated end-diastolic pressure, diminished exercise capacity, increased liver stiffness, and a heightened risk of MACE, including heart transplantation, ventricular assist device implantation, or death [69].

## 5. Future Directions

**Figure 8:**
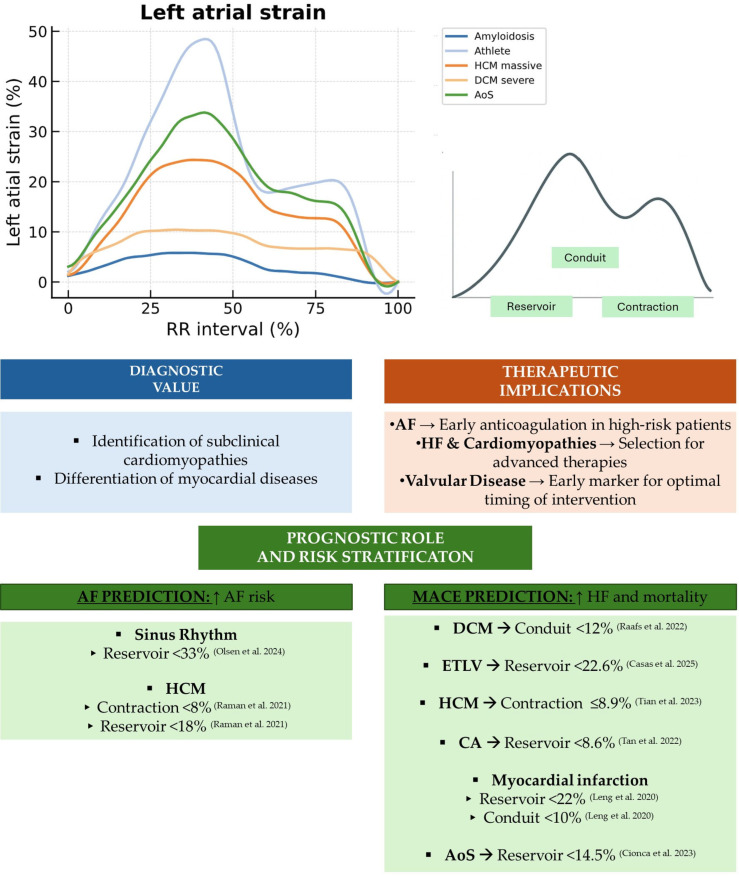
Diagnostic value, prognostic role, and therapeutic implications of LAS evaluated by CMR-FT. The illustration summarizes the utility of LAS, with light green panels indicating threshold values across multiple cardiac pathologies that are relevant for prognostic assessment and risk stratification, as reported in the cited references. LAS, longitudinal left atrial strain; CMR-FT, cardiac magnetic resonance feature tracking; AF, atrial fibrillation; HF, heart failure; MACE, major adverse cardiovascular events; HCM, hypertrophic cardiomyopathy; DCM, dilated cardiomyopathy; ETLV, excessive left ventricular trabeculation; CA, cardiac amyloidosis; AoS, aortic stenosis [33,42,48,54,55,57,63,64].

LAS has emerged as a valuable, non-invasive imaging marker for early diagnosis, risk stratification, and personalized therapeutic decision-making across a broad spectrum of cardiovascular diseases (Table 3, Figure 8).

LAS assessment provides incremental diagnostic value in diagnosing HpEF, aids in detecting subclinical cardiomyopathies, and helps characterize and differentiate myocardial diseases, especially in hypertrophic phenotypes [56].

LAS provides incremental prognostic information independent of traditional parameters such as LAVI, LVEF, and LGE, and helps identify high-risk patients with HF who may benefit from closer clinical surveillance. Its incorporation into guideline-based algorithms may improve patient selection for pharmacological therapies or device interventions, supporting individualized care pathways.

Despite its promising clinical applications, the routine implementation of LAS assessment using CMR-FT remains limited. Key barriers include inter-vendor variability in strain measurements due to differences in post-processing software [28], as well as the lengthy acquisition times inherent to CMR studies and the time required for post-processing atrial strain analysis.

To enable broader clinical implementation, efforts should focus on standardizing imaging protocols, implementing artificial intelligence-guided shorter acquisition protocols, harmonizing strain quantification across software platforms, and developing more efficient and accessible imaging workflows.

Until such solutions are established, serial evaluations should be performed using consistent imaging modalities and software to ensure reproducibility and reliability. Addressing these limitations is essential for the full integration of LA strain into routine cardiovascular practice and for unlocking its diagnostic and prognostic potential.

## 6. Conclusions

The assessment of LA function has become increasingly important in the diagnosis and prognosis of a wide range of cardiovascular diseases. CMR-FT provides a robust, reproducible, and highly accurate method for evaluating LA function. Compared to TTE, CMR-FT offers superior reproducibility and lower interobserver variability.

Given the methodological differences between CMR and TTE, as well as the variability among CMR post-processing software platforms, it is recommended that serial assessments in the same patient be conducted using the same imaging modality and software. This consistency is essential to ensure reliable comparisons over time and to optimize the diagnostic and prognostic value of LAS measurements in both research and clinical settings.

LAS has emerged as a powerful, non-invasive imaging marker that supports early diagnosis, refined risk stratification, and individualized therapeutic decision-making. Integrating LAS assessment into routine clinical CMR protocols, particularly in patients with valvular heart disease or cardiomyopathy, may substantially enhance the diagnostic and prognostic value of cardiac imaging.

Future research should prioritize large-scale, prospective studies with long-term follow-up to evaluate the prognostic value of LAS in individuals with heart disease. Such efforts are essential to validate its clinical utility across diverse patient populations and to support its broader integration into routine practice.

## Figures and Tables

**Table 2:** Consensus-based reference ranges for LA strain by CMR [4].

LA Strain Parameter	Mean	95% CI
LAS reservoir %	34.9	29.6 to 40.2
LAS conduit %	−21.3	−16.6 to −26.1
LAS contraction %	−14.3	−11.8 to −16.8

Adapted from: Sade et al., 2025 [4].

**Table 3:** Clinical applications of atrial strain: diagnostic value and prognostic implications.

Type of Disease	Diagnostic Value	Prognostic Role	Future Clinical Directions
AF	Persistent AF is associated with reduced LAS compared to paroxysmal AF [34].	In sinus rhythm, an LAS reservoir < 33% is associated with an increased risk of AF onset [33].	LAS assessment may support early decision-making regarding the initiation of anticoagulation therapy in patients with stroke risk factors but without documented AF.
HF	LAS assessed during CMR exercise stress may aid in the diagnosis of HFpEF [37]. LAS analysis can aid in distinguishing post-capillary from pre-capillary PH [38].	LAS conduit and reservoir anticipate the risk of HF-related admissions or death [39].	LAS assessment may identify high-risk patients who could benefit from closer monitoring and optimization of medical therapy.
DCM	LAS parameters are commonly reduced in patients with DCM [42].	LAS demonstrates prognostic value, independent of established risk factors, including LVEF and LGE [42,43]. An LAS conduit value < 12% has been associated with an increased risk of MACE [42].	Incorporation of LAS into guideline-based algorithms may identify high-risk patients and support personalized therapeutic decision-making.
ETLV	LAS impairment is commonly observed in patients with excessive ETLV, particularly among those with established cardiomyopathy [48].	An LAS reservoir value < 22.6% has been associated with an increased risk of MACE [48].	LAS could differentiate subclinical cardiomyopathy from physiological hypertrabeculation [48].
HCM	The LAS reservoir is impaired in HCM patients, even in those with normal LA volume and normal left ventricular filling pressures [50,51].	An LAS contraction value ≤ 8.9% has been associated with an increased risk of HF and cardiovascular death [54]. LAS contraction value ≤ 8% and LAS reservoir value ≤ 18% predict new-onset AF [55].	LAS enhances risk stratification [51,53,54], independent of traditional markers such as LA size and LV LGE [53]. Impaired LAS may guide the initiation of prophylactic anticoagulation strategies in this population [55].
CA	LAS is lower in patients with CA compared to those with HCM [56].	Patients with moderate to high amyloid burden exhibit progressive impairments in LAS [57]. An LAS reservoir value < 8.6% has been associated with an increased risk of mortality [57].	Assessment of LAS may offer a valuable, non-invasive method for diagnosing, categorizing, and differentiating CA from other hypertrophic phenotypes [56], while also offering additional prognostic insight [57].
Myocardial infarction	LAS reliably identifies the presence and severity of diastolic dysfunction and is also correlated with infarct size [62].	LAS reservoir (≤22%) and LAS conduit strain (≤10%) have been identified as predictors of MACE [63].	LAS could provide incremental prognostic value over established outcome predictors, such as LVEF, microvascular obstruction, and infarct size quantified by LGE [63].
AoS	Patients with AoS often exhibit impaired LAS parameters [64].	An LAS reservoir value < 14.5% is a predictor of MACE [64].	LA functional abnormalities may precede and potentially predict LV remodeling in patients with AoS.
		TAVR is associated with improvements in LA functional parameters, indicating a reversal of myocardial remodeling and restoration of LA function [65].	Atrial dysfunction could serve as an early indicator for the timing of valvular intervention.

AF, atrial fibrillation; LAS, longitudinal left atrial strain; HF, heart failure; CMR, cardiac magnetic resonance; HFpEF, heart failure with preserved ejection fraction; PH, pulmonary hypertension; LA, left atrial; DCM, dilated cardiomyopathy; LVEF, left ventricular ejection fraction; LGE, late gadolinium enhancement; MACE, major adverse cardiovascular events; ETLV, excessive left ventricular trabeculation; HCM, hypertrophic cardiomyopathy; LV, left ventricle; CA, cardiac amyloidosis; AoS, aortic stenosis; TAVR, transcatheter aortic valve replacement.

## Data Availability

No new data were generated during this study.

## Abbreviations

The following abbreviations are used in this manuscript:

AF	atrial fibrillation
AFD	Anderson–Fabry disease
AL	amyloid light-chain
AoS	aortic stenosis
AR	atrial reversal
ATTR	amyloid transthyretin
AUC	area under the curve
AV	atrioventricular
CA	cardiac amyloidosis
CMR	cardiac magnetic resonance.
CMR-FT	cardiac magnetic resonance feature tracking
D	diastolic
DCM	dilated cardiomyopathy
ECG	electrocardiogram
ECV	extracellular volume
ED	end-diastolic
ETLV	excessive left ventricular trabeculation
FT	feature tracking
HCM	hypertrophic cardiomyopathy
HF	heart failure
HFpEF	heart failure with preserved ejection fraction
HFrEF	heart failure with reduced ejection fraction
LA	left atrium
LAS	longitudinal left atrial strain
LAVI	left atrial volume index
LGE	Late gadolinium enhancement
LV	left ventricle
LV GLS	left ventricular global longitudinal strain
LVEF	left ventricular ejection fraction
MACE	major adverse cardiovascular events
MVO	mitral valve opening
NT-proBNP	N-terminal pro–B-type Natriuretic Peptide
NYHA	New York Heart Association
PH	pulmonary hypertension
S	systolic
SSc	systemic sclerosis
STEMI	ST-segment elevation myocardial infarction
TAVR	Transcatheter aortic valve replacement
TTE	transthoracic echocardiography
